# Depression prediction and key factors: A comparative analysis of logistic regression and machine learning models

**DOI:** 10.1371/journal.pone.0354668

**Published:** 2026-08-27

**Authors:** Kripa Josten, Vennila Jaganathan

**Affiliations:** 1 Research Scholar, Manipal College of Health Professions, Manipal Academy of Higher Education, Manipal, Karnataka, India; 2 Associate Professor, Manipal College of Health Professions, Manipal Academy of Higher Education, Manipal, Karnataka, India; Kalinga Institute of Medical Sciences, INDIA

## Abstract

**Objectives:**

Factors associated with depression were explored in this study through logistic regression, and predictive performance was compared with various Machine Learning models.

**Methods:**

WHO SAGE India wave 2 data were used with depression as the outcome variable. and predictors were sociodemographic, health, and psychosocial variables. Descriptive analysis and Logistic regression were estimated. Random Forest, XGBoost, Support Vector Machine, Logistic Regression, Bagging, Decision Tree, Naïve Bayes, Ridge Logistic Regression, Neural Networks, and K Nearest Neighbors are the ten Machine Learning algorithms that were used. Performance measures consisted of accuracy, Area Under Curve, precision, recall, F1 score, Hamming loss, Jaccard score, and Matthew’s correlation coefficient. Random Forest and XGBoost were used to assess feature importance.

**Results:**

Depression was also more prevalent among younger adults, women, and individuals with poor self-rated health, stress, and sleep disturbances. Logistic regression revealed age and feeling low or sad as a factor (p = 0.008, p = 0.021). Most models demonstrated only moderate discriminative ability, with the AUC below 0.70, with better-performing models being Ridge regression (AUC = 0.716) and Random Forest (AUC = 0.713). Feature importance universally identified age, perception of health, quality of life, and depressive symptoms as important predictors.

**Conclusions:**

Logistic regression provides interpretability, and Machine Learning increases predictive accuracy. Combining both can enhance depression prediction and screening in public health practice.

## Background

Depression contributes significantly to the global burden of disease and is a leading cause of global disability [1]. The World Health Organisation (WHO, 2023) estimated that more than 280 million people are affected across various age groups, amounting to approximately 3.8% of the global population, which includes 5% of adults and 5.7% of elderly aged over 60 years [2,3]. The Global Burden of Disease (GBD) study (2019) estimated that depressive disorders accounted for over 49.5 million disability-adjusted life years (DALYs) globally, ranking among the top five causes of years lived with disability (YLDs) [4]. In addition to affecting everyday functioning and quality of life, depression significantly raises the chances of chronic illnesses, premature mortality, and suicide, which claims the lives of about 7,00,000 individuals annually globally [5–7].

The prevalence of depression is comparatively high in India [8]. The National Mental Health Survey (NMHS, 2015–16) reported a current prevalence of 2.7% and a lifetime prevalence of 5.25% among adults [9]. Estimates from the GBD study (2019) suggest that nearly 197 million Indians were living with mental disorders, with depressive disorders contributing a significant proportion and a point prevalence of 3.3–3.5% [4]. The results of the National Family Health Survey-5 (2019–21) indicate that mental health issues are particularly prevalent among women and adolescents. In India, nearly one in every 20 individuals experiences depression during their lifetime, with higher incidence reported in urban populations, among females, and socioeconomically disadvantaged groups [10,11]. The burden is increased by underdiagnosis, stigma, and limited access to mental health services, emphasising the urgent need for prompt identification and management as a key public health concern [12–14].

Machine learning (ML) has rapidly evolved in healthcare research. Applications extend over various domains, such as hypertension [15], cancer prognosis [16], chronic kidney disease [17], etc, where ML models such as support vector machines (SVM), random forests, and gradient boosting have achieved robust predictive accuracy. Recent studies emphasise the importance of ML models in predicting depression. While the traditional approach to investigating depression offers interpretability and statistical inference, making it relevant for public health decision-making [18,19]. For example. Studies have demonstrated across various populations that self-rated health, marital status, and psychosocial variables are associated with depression. However, they may face limitations in capturing complex, nonlinear interactions between predictors, which are often present in mental health data.

ML techniques, such as Random Forests, Support Vector Machines (SVMs), and gradient boosting methods, have emerged as powerful tools in predictive modelling. Lee and Kim (2022) applied ML to predict depression among the hypertensive population in the U.S ^20^. In addition, other studies from around the world have shown that ensemble and hybrid ML approaches better capture complex psychosocial and clinical interactions in depression prediction. Exploiting most large datasets and subtle relationships, ML models are capable of outperforming conventional regression models in classification problems [21–23]. Nevertheless, a common critique is that they may compromise interpretability for predictive accuracy, posing challenges for clinical translation.

At the global level, many studies have applied ML to predict depression in various populations (e.g., hypertensive, elderly, etc.); however, evidence from India remains scarce. Hence, integrating traditional statistical methods with ML approaches could lead to a more holistic knowledge of depression. While logistic regression can identify interpretable and statistically significant predictors, ML models can evaluate predictive efficacy and underscore the possibilities for scalable screening solutions [24].

Therefore, this study aims to examine the factors associated with depression using logistic regression and to compare its predictive ability with a range of ML algorithms. By combining different methods, we aim to emphasize both the factors contributing to depression and the possible contribution of ML in improving mental health forecasting and early identification.

## Methodology

### Study design and participants

This study utilised cross-sectional data collected by WHO SAGE WAVE 2 in India across six states [25]. The initial dataset included 11,818 participants aged 18–101 years. For the present analysis, only individuals aged 18–59 years were included to focus on the working-age population, as factors associated with depression in older adults may differ due to retirement, chronic disease burden, and age-related health transitions. Participants were included in the analysis if they had complete information on the outcome variable and all selected predictors. After applying the age restriction and excluding cases with missing data on key variables, the final analytic sample consisted of 429 participants. The outcome variable was depression, which was defined based on self-reported physician diagnosis using the WHO SAGE questionnaire item Q4040: “Since we last spoke, have you been told by a doctor that you have depression?” Responses were coded as 1 = Yes and 2 = No. Sociodemographic, clinical, and psychosocial factors associated with depression were considered, which included age, gender, marital status, occupational history, self-rated health, sleep problems, anxiety, concentration difficulties, stress, suicidality, and perceived quality of life.

### Ethical approval

This study used the secondary data collected by the WHO in collaboration with country research organizations. The SAGE study was approved by the Ethics Review Committee, WHO, Geneva, Switzerland and the Institutional Review Board, International Institute of Population Sciences, Mumbai, India.

### Statistical analysis

Statistical analysis was done using R software. Descriptive statistics were used to summarize the distribution of sociodemographic and clinical variables. Continuous variables were reported as medians and interquartile ranges (IQR) since the normality assumption was violated, while categorical variables were presented as frequencies and percentages.

To determine the significant predictors of depression, a binary logistic regression model was used. Results are presented as odds ratios (OR) with corresponding 95% confidence intervals (CI) and p-values. Statistical significance was considered at p < 0.05.

### Machine learning models

Prior to creating the ML models, the dataset was preprocessed meticulously. The dataset was divided into training (80%) and testing (20%) subsets through stratified sampling to maintain the ratio of depressed and non-depressed participants within both sets. The same train-test was used for all the ML models to ensure fair comparison across them. 5-fold cross-validation was utilized within the training data to optimize model hyperparameters and prevent overfitting.

Ten ML models were selected to provide a comprehensive comparison across algorithmic families and to find the most suitable algorithm for the data. The selection included a mix of linear, nonlinear, ensemble-based, and probabilistic approaches enabling assessment of complex interactions and potential class imbalance within the dataset. Logistic regression was included as a baseline model, while Ridge Logistic Regression introduced L2 regularization to improve generalizability. Decision Tree models were trained with the Gini index as the splitting measure, using pruning to reduce overfitting. Ensemble techniques such as Random Forest, which averages many decision trees learned from bootstrapped samples with randomly selected features, and Bagging, take the average of predictions from weak learners to minimise variance. Gradient boosting was exemplified by XGBoost, which sequentially constructs trees using gradient descent optimization with regularization [26,27].

Among the nonlinear models, SVM with a radial basis function kernel was used to capture complex boundaries, while K-Nearest Neighbours (KNN) was used as a distance-based classifier with the optimal number of neighbours determined through cross-validation. A Naïve Bayes classifier was added to simulate conditional probabilities under independence assumptions, and an Artificial Neural Network (ANN) was developed with one hidden layer using (Rectified Linear Unit) activation and a sigmoid output function optimized using backpropagation and the Adam optimizer.

Model hyperparameters were optimized using five-fold cross-validation within the training dataset using a grid-search strategy to identify parameter combinations that maximized predictive performance. Key hyperparameters for each ML model were tuned during this process to improve model generalizability and reduce overfitting. Details of the hyperparameters considered for each algorithm are provided in S1 Table.

The performance of all models was assessed on the testing dataset using a wide set of evaluation metrics. Accuracy was reported as the proportion of correctly classified cases, while the area under the curve (AUC) quantified the ability of the model to discriminate between depressed and non-depressed participants across thresholds. Precision, recall (sensitivity), and F1 score were used to evaluate the balance between false positives and false negatives. Moreover, hamming loss and Jaccard score provided alternative measures of classification error and overlap, respectively, and the Matthews Correlation Coefficient (MCC) was included as a robust performance measure for imbalanced datasets. For ease of interpretation, results were tabulated and further depicted via bar charts for accuracy, AUC, F1 measure, and MCC for models.

### Handling of class imbalance

Depression prevalence in this sample was relatively low (31 of 429 participants, ~ 7.2%), resulting in class imbalance between depressed and non-depressed groups. To preserve the class distribution, stratified sampling was used during the train-test split. Model performance was evaluated using multiple metrics beyond accuracy, including recall (sensitivity), F1 score, and the Matthews Correlation Coefficient (MCC), which provides a more balanced measure of performance in imbalanced datasets. Resampling approaches such as SMOTE were not applied in order to preserve the original data distribution and allow consistent comparison across models.

## Results

### Descriptive characteristics

Table 1 presents the descriptives of sociodemographic, clinical, and psychosocial variables classified by depression status. Median age was lower in participants with depression (48 years [IQR: 42–52.5]) compared to those without depression (53 years [IQR: 48–56]). Prevalence of depression among females was higher (4.9%) compared with males (2.3%). Depression was highest among widowed individuals (1.4%) and those currently married (5.1%). Participants who had ever worked reported higher rates of depression (5.4%) compared with those who never worked (1.9%).

**Table 1:** Descriptive statistics.

Variables		Depression	
		Yes N (%)	No N (%)
Age	Median [IQR]	48 [42,52.5]	53 [48,56]
Gender (q1009)	Female	21 (4.9)	265 (61.8)
	Male	10 (2.3)	133 (31.0)
Current Marital Status (q1012)	Currently Married	22 (5.1)	300 (69.9)
	Currently not Married	9 (2.1)	98 (22.8)
Ever worked (q1501)	Yes	23 (5.4)	280 (65.3)
	No	8 (1.9)	118 (27.5)
Rate your health (q2000)	Good	8 (1.9)	114 (26.6)
	Moderate	15 (3.5)	194 (45.2)
	Bad	8 (1.9)	90 (21.0)
Feeling sad/low/depression (q2018)	Mild	12 (2.8)	169 (39.4)
	Moderate	5 (1.2)	97 (22.6)
	Severe	13 (3.0)	52 (12.1)
	Extreme/ Cannot do	0	3 (0.7)
	None	1 (0.2)	77 (17.9)
Sudden loss of feeling (q4013)	Yes	3 (0.7)	34 (7.9)
	No	28 (6.5)	364 (84.8)
Problems sleeping (q4049)	Yes	23 (5.4)	240 (55.9)
	No	8 (1.9)	158 (36.8)
Difficulties concentrating (q4051)	Yes	12 (2.8)	162 (37.8)
	No	19 (4.4)	236 (55.0)
Anxious (q4053)	Yes	23 (5.4)	263 (61.3)
	No	8 (1.9)	135 (31.5)
Suicidality (q4059)	Yes	3 (0.7)	25 (5.8)
	No	28 (6.5)	373 (86.9)
Rate the overall quality of life (q7009)	Good	9 (2.1)	95 (22.1)
	Moderate	12 (2.8)	223 (52.0)
	Bad	10 (2.3)	77 (17.9)
	Don’t know	0	3 (0.7)
Felt tensed or stressed (q7505)	Yes	18 (4.2)	157 (36.6)
	No	13 (3.0)	241 (56.2)

Health and psychosocial factors were also closely linked with depression status. Prevalence of depression was the highest in individuals with “moderate” self-assessment of health, those with sleep issues (5.4%), anxiety (5.4%), problems concentrating (2.8%), or stress (4.2%). Similar patterns were observed for suicidality and acute loss of sensation, although the absolute number was low.

### Logistic regression findings

The binary logistic regression results are presented in Table 2. Age was significantly associated with depression, with increasing age reducing the odds of depression (OR = 0.944, 95%, *p* = 0.008). In addition, Feeling sad/low/depressed was also found to be a significant predictor (OR = 3.082, 95%, *p* = 0.021).

**Table 2:** Logistic regression for depression predictors.

Variables	Categories	Odds Ratio (OR)	95% CI (Lower, Upper)	p-value
Age (q1011)	Continuous	0.944	(0.90, 0.98)	0.008*
Gender (q1009)	Female (Ref)	–	–	–
	Male	0.971	(0.38, 2.43)	0.950
Current Marital Status (q1012)	Currently Married (Ref)	–	–	–
	Currently not married	1.106	(0.44,2.71)	0.827
Ever worked (q1501)	No	–	–	–
	Yes	1.457	(0.54,3.89)	0.452
Rate your health (q2000)	Good	1.046	(0.31,3.42)	0.941
	Moderate	1.034	(0.38,2.80)	0.947
	Bad (Ref)			
Feeling sad/low/depressed (q2018)	Mild (Ref)	–	–	–
	Moderate	0.695	(0.23,2.09)	0.519
	Severe	3.082	(1.18,7.98)	0.021*
	None	0.186	(0.02,1.49)	0.113
	Extreme/ Cannot do	NE		
Sudden loss of feeling (q4013)	No (Ref)	–	–	–
	Yes	1.131	(0.29, 4.36)	0.859
Problems sleeping (q4049)	No (Ref)	–	–	–
	Yes	1.634	(0.62, 4.26)	0.316
Difficulties concentrating (q4051)	No (Ref)	–	–	–
	Yes	0.540	(0.22, 1.29)	0.166
Anxious (q4053)	No (Ref)	–	–	–
	Yes	1.041	(0.39, 2.74)	0.935
Suicidality (q4059)	No (Ref)	–	–	–
	Yes	1.303	(0.32, 5.20)	0.708
Rate overall quality of life (q7009)	Good	1.251	(0.42,3.71)	0.687
	Moderate	0.584	(0.22,1.54)	0.279
	Bad (ref)			
	Don’t know	NE		
Felt tensed/stressed (q7505)	No (Ref)	–	–	–
	Yes	1.82	(0.79, 4.19)	0.077

*Statistically Significant

*NE: Not estimable due to sparse observations or quasi-complete separation in the logistic regression model.*

Other predictors, such as gender, marital status, employment status, sleep problems, suicidality, stress, and concentration difficulties, did not show statistical significance but had higher odds. Notably, wide confidence intervals were observed for several predictors, suggesting instability due to small subgroup sizes.

### Machine learning model performance

**Fig 1:**
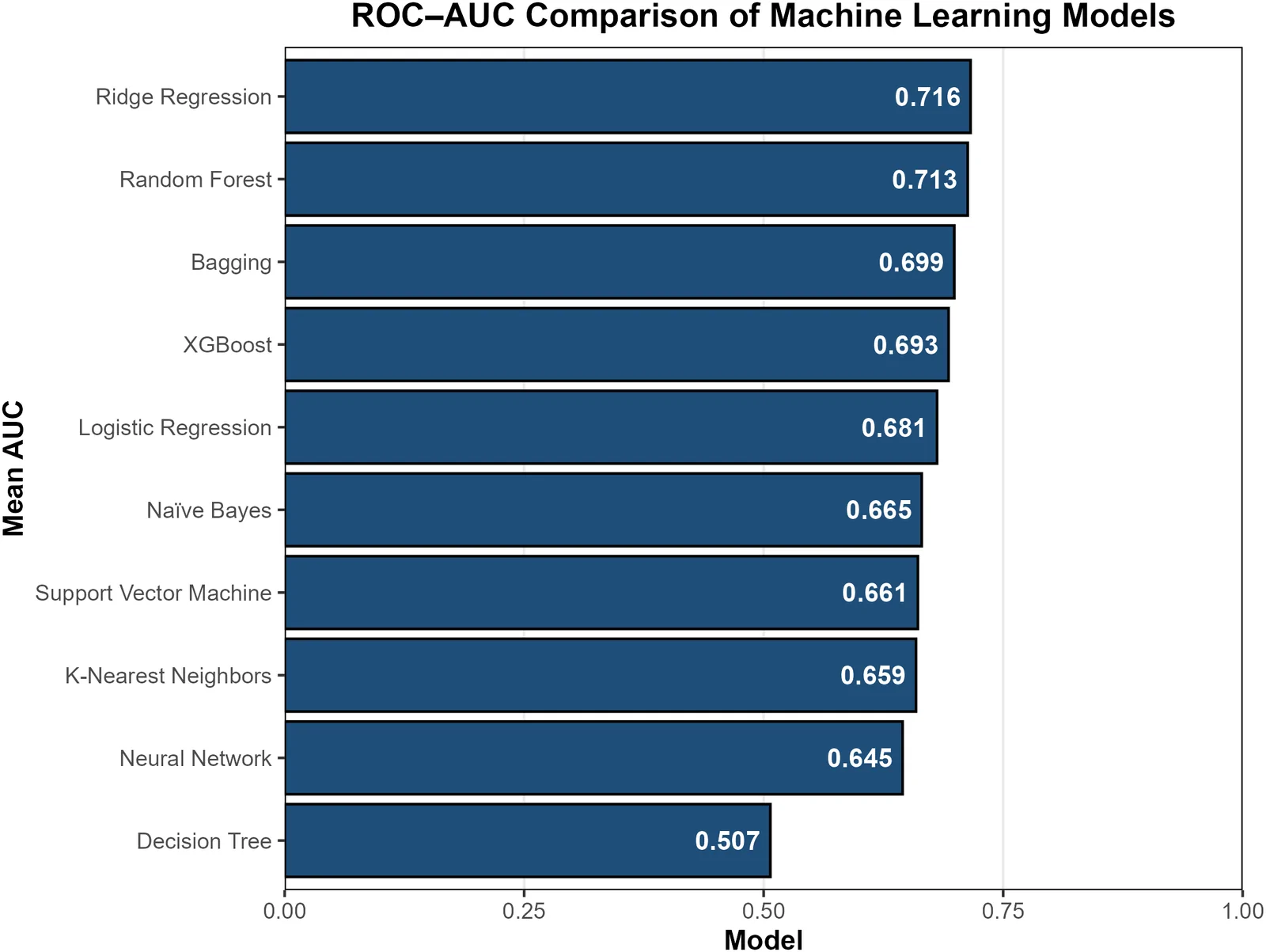
Comparison of the predictive performance of ML models for detecting depression in the study population.

**Fig 2:**
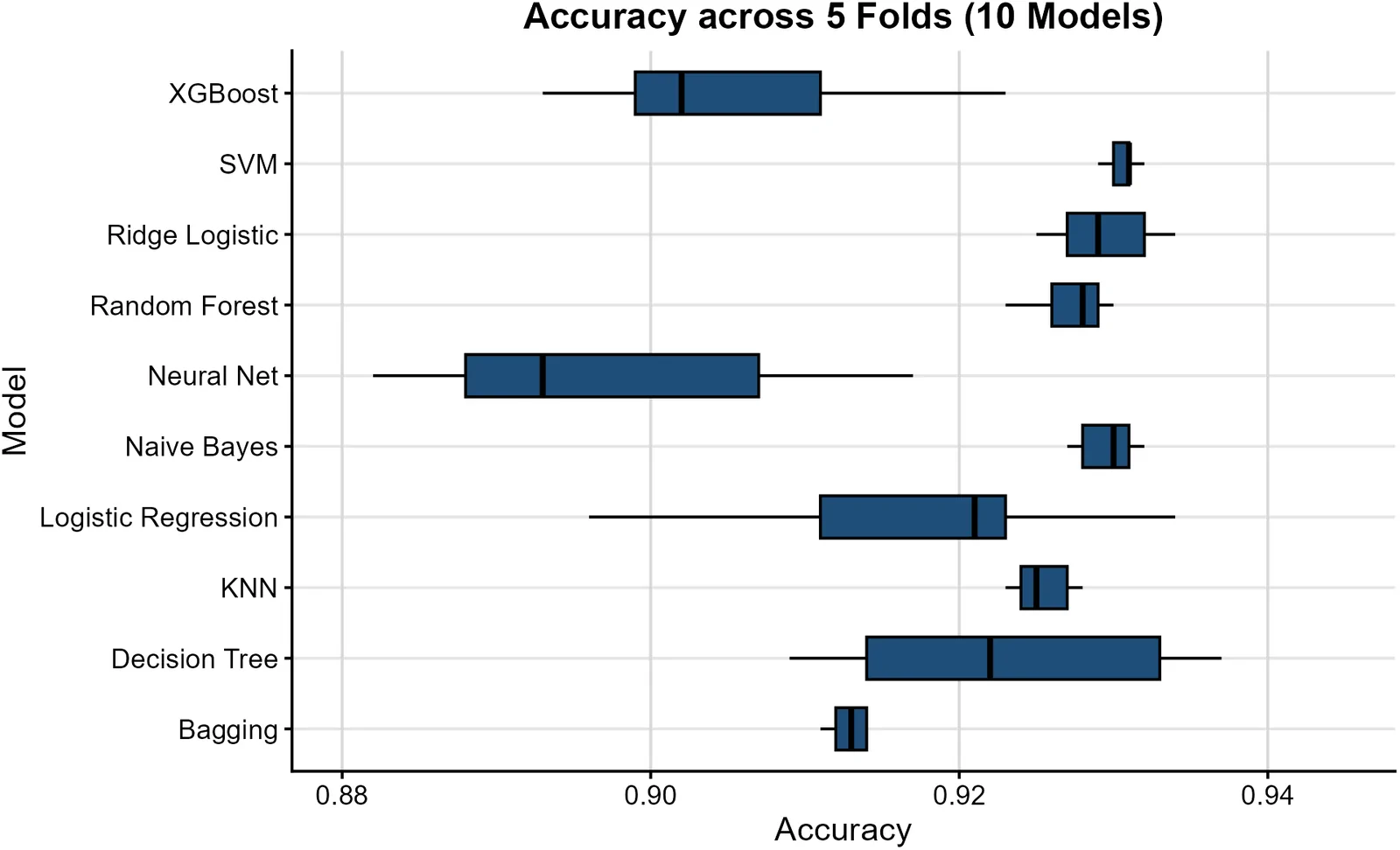
Distribution of classification accuracy across five-fold cross-validation for ML models predicting depression in the study population.

**Fig 3:**
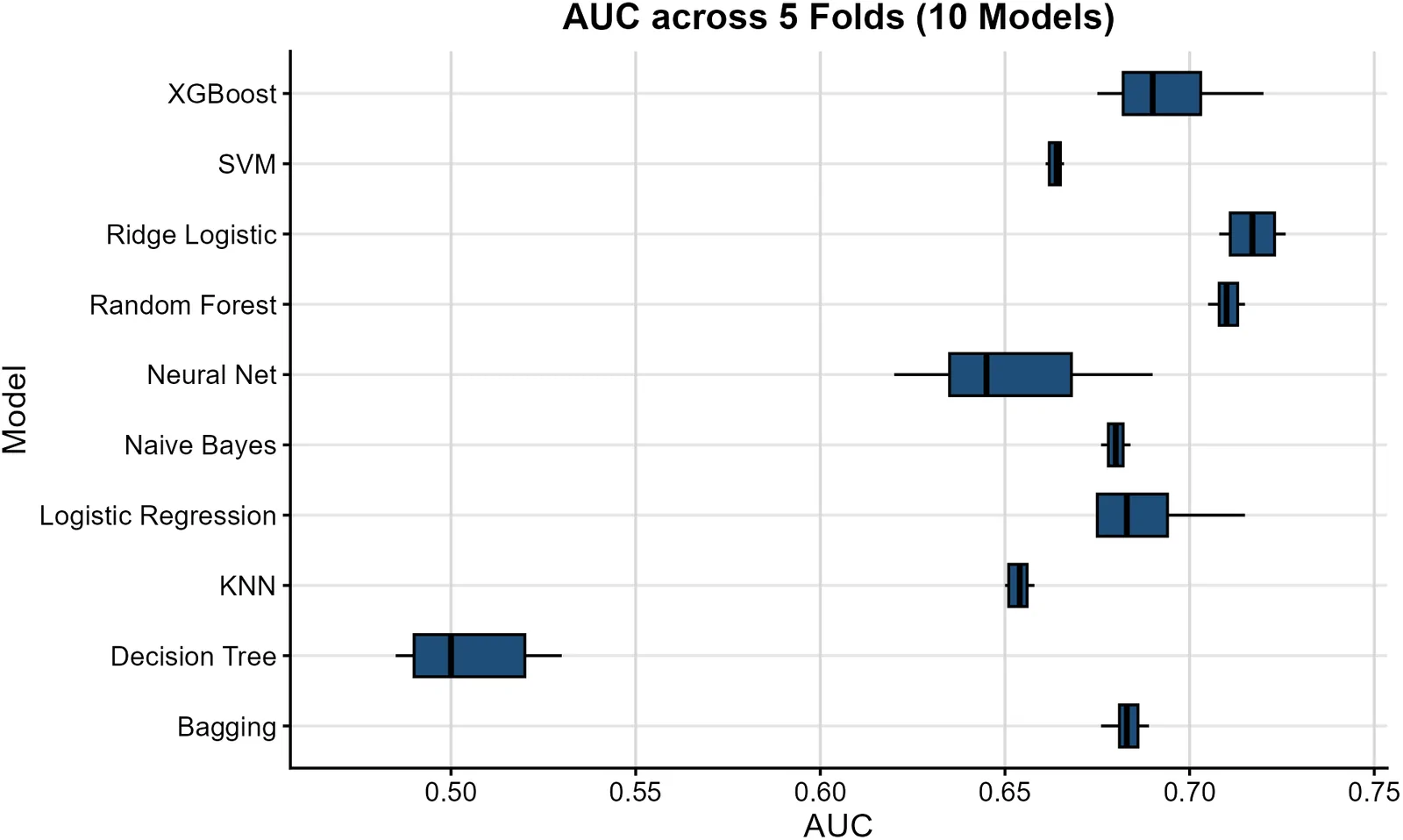
Distribution of AUC across five-fold cross-validation for ML models predicting depression in the study population.

**Fig 4:**
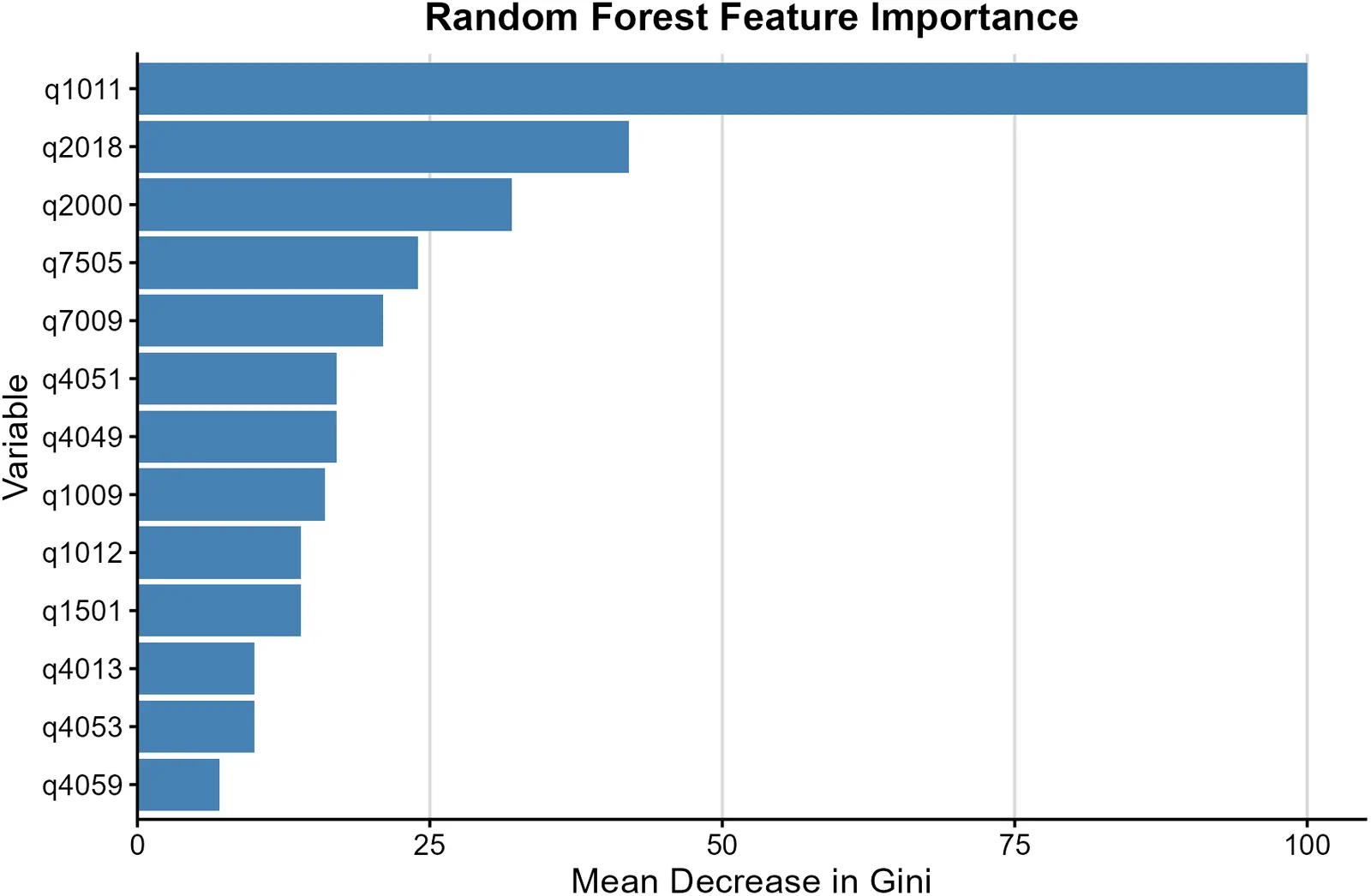
Feature importance derived from Random Forest model predicting depression in the study population.

**Fig 5:**
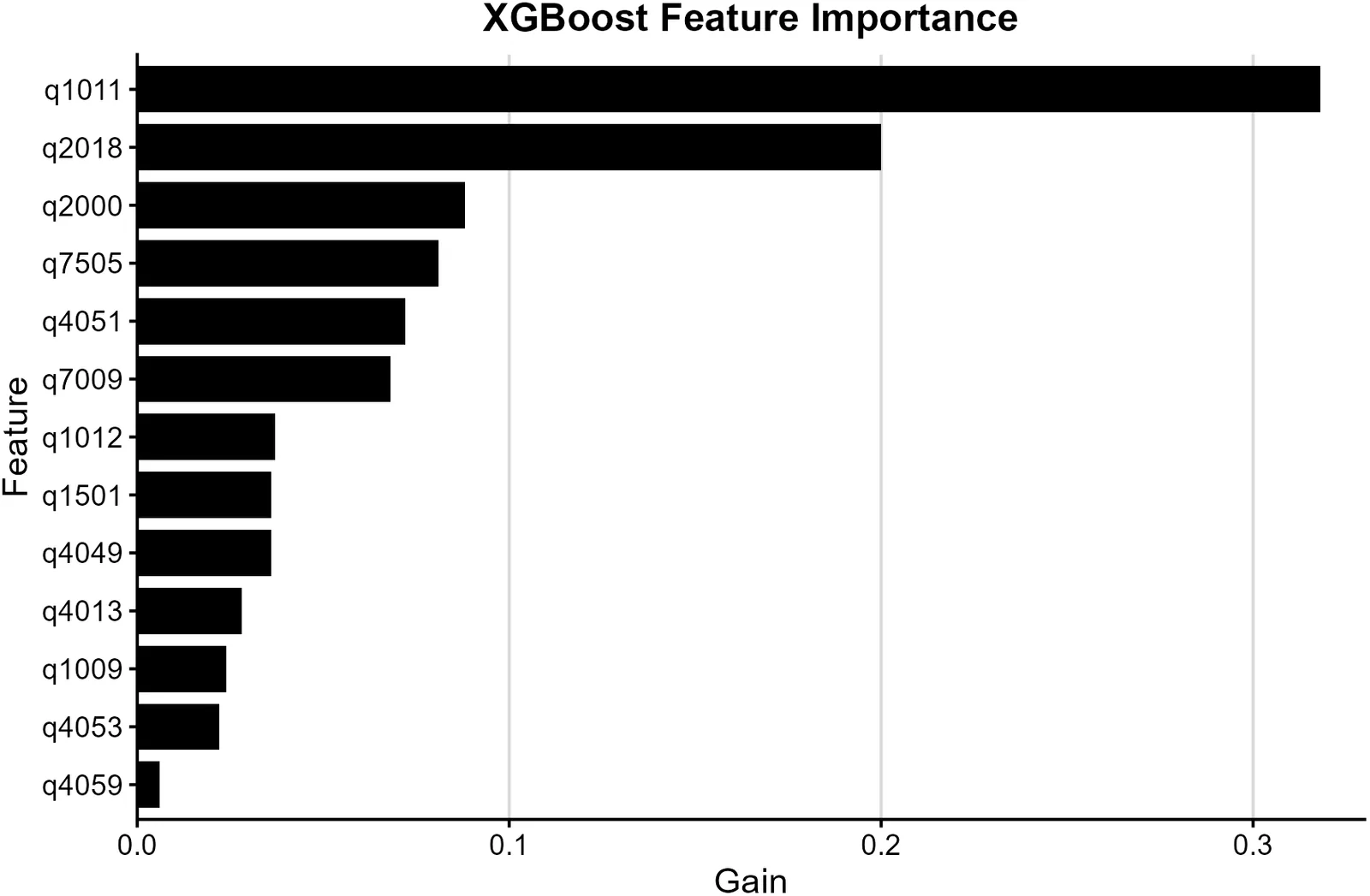
Feature importance derived from XGBoost model predicting depression in the study population.

The predictive performance of the ten ML models is illustrated in Figs 1–5 and the primary model performance metrics are presented in Table 3.Additional metrics such as precision, Hamming loss, and Jaccard score, are provided in the S2 Table.

**Table 3:** Results of multiple ML models.

Model	Mean Accuracy	Mean AUC	Recall	F1 Score	MCC
Random Forest	0.928	0.713	0.920	0.925	0.710
XGBoost	0.906	0.693	0.880	0.818	0.671
SVM	0.928	0.661	0.940	0.930	0.700
Logistic Regression	0.916	0.681	0.910	0.910	0.620
Bagging	0.921	0.699	0.920	0.915	0.600
Decision Tree	0.925	0.507	0.930	0.925	0.590
Naive Bayes	0.923	0.665	0.930	0.920	0.580
Ridge Logistic	0.928	0.716	0.930	0.925	0.570
Neural Net	0.895	0.645	0.890	0.885	0.480
KNN	0.928	0.659	0.930	0.920	0.560

Fig 1 illustrates the area under the Receiver Operating Characteristic (ROC) curve for the various ML models, with most showing only moderate discriminatory ability despite high accuracy. Ridge regression achieved the highest AUC (0.716), followed by Random Forest (0.713). Logistic Regression had an AUC of 0.681, and Decision Tree was the lowest at 0.507.

Accuracy across five-fold cross-validation is illustrated in Fig 2, where Random Forest, SVM, Ridge Logistic, and KNN achieved the highest accuracy (0.928), followed closely by Decision Tree and Naïve Bayes (~0.923–0.925). Logistic Regression (0.916) demonstrated moderate performance, whereas the Neural Network achieved the least accuracy (0.895). The boxplot represents the IQR, with whiskers indicating the spread of accuracy values across folds.

Fig 3 presents the Distribution of AUC across five-fold cross-validation. Boxplots show the variability of AUC values across five folds for ten models. Random Forest, XGBoost, and SVM consistently achieved higher median AUCs with relatively narrow ranges, while Logistic Regression, Naïve Bayes, and Neural Net exhibited lower and more variable AUC performance. Overall, these findings suggest that ensemble-based models demonstrated comparatively better discrimination, although the overall predictive performance remained moderate.

Random Forest (Fig 4) and XGBoost (Fig 5) used the feature importance plots to highlight the most influential predictors of depression. For Random Forest Predictor importance is measured using the mean decrease in the Gini index, where higher values indicate greater contribution of the variable to the model’s classification performance. While for XGBoost model importance is measured using the gain metric, representing the relative contribution of each predictor variable to improving model performance. In both models, age emerged as one of the most significant predictors, underscoring its protective role against depression as age increases. Self-rated health and depressive symptoms were other predictors, implying that subjective health perception and emotional well-being are critical in depression severity classification. The convergence of both ensemble-based models on similar predictors strengthens the evidence for the multifactorial predicators of depression and demonstrates the utility of ML in identifying complex patterns beyond traditional regression.

In general, descriptive analyses indicated depression to be more prevalent in younger individuals, women, widows, and those with psychosocial or health problems. Logistic regression identified age and those who are feeling low/sad/depressed as key predictors for depression, whereas other variables were not statistically significant. ML algorithms, especially Ridge regression and Random Forests, achieved better predictive performance across other metrics; however, the overall AUC values indicate moderate discrimination.

## Discussion

This study examined the factors associated with depression and evaluated the predictive performance of multiple ML models in comparison with traditional logistic regression. The descriptive analysis indicated that depression was more prevalent among younger individuals, particularly females, those with poor self-rated health, and participants reporting psychosocial difficulties such as sleep problems, anxiety, and stress [28,29]. These results align with prior studies conducted in India [9] and globally [20,30], which have highlighted the role of sociodemographic and psychosocial factors in determining susceptibility to depression.

The logistic regression analysis identified age as a significant predictor, with increasing age being associated with lower odds of depression. This is consistent with the evidence from epidemiological studies suggesting that depression often peaks in midlife and declines in older age, possibly due to resilience or generational differences [3,31]. Indian evidence from the National Mental Health Survey (2015–16) and community-based studies [32,33] similarly reports higher vulnerability among younger and middle-aged adults, women, and individuals with chronic illnesses. Additionally, participants with severe depressive symptoms had higher odds compared with mild symptoms, which is consistent with clinical findings where feeling emotionally low or sad is a key indicator of underlying depression [34].

Although psychosocial factors such as sleep problems, suicidality, and stress showed elevated odds ratios, their associations did not reach statistical significance in this dataset. The wide confidence intervals suggest instability, likely due to small subgroup sample sizes. Nevertheless, these trends are consistent with established literature linking sleep disturbances, suicidal ideation, and stress with depression [4].

The ML analysis provided insights into predictive performance. Ridge Regression and Random Forest demonstrated the strongest overall classification ability, while Random Forest and Support Vector Machine achieved he highest accuracy with accuracy and F1 scores exceeding 0.92. These results suggest that nonlinear and ensemble-based approaches can capture complex patterns not easily modelled using regression alone, which is consistent with previous studies done in hypertension, cancer, and mental health, where SVM, XGBoost and Random Forest frequently outperform other models [15,16,20]. However, despite strong performance in accuracy and F1 score, most models achieved only moderate discrimination with AUC values around 0.70. This discrepancy, prevalent in imbalanced datasets, suggests that although models were effective at identifying the majority class, their ability to discriminate between depressed and non-depressed individuals across thresholds was limited. Global studies applying ML to psychiatric prediction similarly report challenges in attaining high AUCs, reflecting the multifactorial aetiology of depression [35,36].

The relatively low prevalence of depression in the dataset also introduces class imbalance. Hence classification accuracy may appear high if models correctly predict the majority group while misclassifying a substantial proportion of the minority (depressed) class. Although the F1 score balances precision and recall, it may still be influenced by class distribution. Therefore, additional metrics, such as the MCC, were reported, as it incorporates all elements of the confusion matrix and provides a more reliable measure of model performance in imbalanced classification settings. The inclusion of multiple performance metrics allows for a more comprehensive assessment of predictive validity beyond accuracy alone.

The comparison of logistic regression and ML underscores the relationship between interpretability and predictive performance. Logistic regression remains advantageous for identifying interpretable, statistically significant predictors that directly influence public health strategies and policies. On the contrary, ML methods offer higher classification accuracy and stability, making them beneficial for screening systems and for scalable prediction of factors associated with depression. However, the goal was exploratory in both contexts, that is, to examine the potential associations and to evaluate the predictors within the dataset. Importantly, feature importance analysis highlighted age, self-rated health, and quality of life as the top predictors, closely aligning with established predictors reported in both Indian and global literature [37–39].

Overall, these findings emphasise that an integrated approach, leveraging the interpretability of statistical models and the predictive power of ML, may offer the most practical value for mental health research and practice. Such integration could strengthen early detection tools, improve community-level screening, and guide targeted interventions.

### Strengths and limitations

The main advantage of this study is that it uses both conventional and contemporary methods, allowing for a comprehensive understanding of depression prediction. Various ML algorithms were systematically evaluated against diverse performance metrics, yielding strong evidence of their comparative strengths and weaknesses

However, the cross-sectional nature of the data restricts causal inference, as associations cannot establish temporality. Secondly, depression was defined as a self-reported physician diagnosis rather than a clinical assessment, which may have recall or reporting bias. In addition, the analysis relied on a nationally representative dataset. Some predictor categories contain a very small number of observations, leading to unstable estimates and wider confidence intervals that may diminish statistical power. Hence, these results should be interpreted with caution. Furthermore, while ML models achieved high accuracy, their moderate AUC values highlight potential issues of class imbalance that could have affected classification performance. Additionally, the ML analysis was performed using publicly available R packages with standard computing resources. More advanced deep learning models and computationally intensive optimization techniques were not implemented due to limited computational capacity. Future approaches should involve more extensive, longitudinal datasets and apply advanced techniques like resampling or cost-sensitive learning to tackle imbalance

## Conclusion

This study provides important insights into the predictors of depression by integrating traditional logistic regression with advanced ML approaches. ML models demonstrated superior classification performance compared to logistic regression, though their discriminatory power, as measured by AUC values, remained moderate. These findings suggest that ML models can uncover complex nonlinear patterns while statistical models provide interpretability, making their integration a promising approach for public health applications. Future studies utilising longer-term data and larger, more balanced datasets are necessary to improve discrimination, enhance predictive models, and convert these methods into feasible plans for reducing the prevalence of depression in India and elsewhere.

## Future directions

This study demonstrates the potential of predicting depression using both logistic regression and various ML models. Future research should focus on validating models using larger and more heterogeneous, mainly primary datasets to ensure the generalizability. A subsequent step lies in bridging the gap between methodological advances and clinical usability. Eventually, the success will not only depend on accuracy but also on the ability of predictive models to improve decision-making. Personalise strategies and operate within a stipulated time. By addressing these considerations, predictive modelling can move closer to supporting more mental health interventions

## Supporting information

S1 Table Hyperparameter tuning for the ML models. (PDF)

S2 Table Supplementary performance metrics (precision, Hamming loss, and Jaccard score) for machine learning models used to predict depression in the study population. (PDF)

## Abbreviations

AUC: Area Under the Curve
CI: Confidence Interval
DALY: Disability-Adjusted Life Years
F1 Score: Harmonic Mean of Precision and Recall
GBD: Global Burden of Disease
KNN: K-Nearest Neighbors
MCC: Matthews Correlation Coefficient
ML: Machine Learning
NFHS: National Family Health Survey
NMHS: National Mental Health Survey
OR: Odds Ratio
ROC: Receiver Operating Characteristic
SVM: Support Vector Machine
WHO: World Health Organization
YLD: Years Lived with Disability
ReLU: Rectified Linear Unit.

## References

[1] Reddy MS. Depression: the disorder and the burden. Indian J Psychol Med. 2010;32(1):1–2. doi: 10.4103/0253-7176.70510 21799550 PMC3137804

[2] Evans-Lacko S, Aguilar-Gaxiola S, Al-Hamzawi A, Alonso J, Benjet C, Bruffaerts R, et al. Socio-economic variations in the mental health treatment gap for people with anxiety, mood, and substance use disorders: results from the WHO World Mental Health (WMH) surveys. Psychol Med. 2018;48(9):1560–71. doi: 10.1017/S0033291717003336 29173244 PMC6878971

[3] World Health Organization. Depressive disorder (depression). 2023.

[4] GBD 2019 Mental Disorders Collaborators. Global, regional, and national burden of 12 mental disorders in 204 countries and territories, 1990–2019: a systematic analysis for the Global Burden of Disease Study 2019. Lancet Psychiatry. 2022;9(2):137–50. doi: 10.1016/S2215-0366(21)00395-3 35026139 PMC8776563

[5] Remes O, Francisco J, Templeton P. Biological, psychological, and social determinants of depression: a review of recent literature. Brain Sci. 2021;11(12). doi: 10.3390/brainsci11121633 PMC8699555 34942936

[6] Sadek J, Diaz-Piedra B, Saleh L, MacDonald L. A narrative review: suicide and suicidal behaviour in older adults. Front Psychiatry. 2024;15:1395462. doi: 10.3389/fpsyt.2024.1395462 38800059 PMC11117711

[7] Tong Y, Wang Q, Wang X, Xiang Y, Cheng L, Hu X, et al. A scoping review of functional near-infrared spectroscopy biomarkers in late-life depression: depressive symptoms, cognitive functioning, and social functioning. Psychiatry Res Neuroimaging. 2024;341:111810. doi: 10.1016/j.pscychresns.2024.111810 38555800

[8] Arvind BA, Gururaj G, Loganathan S, Amudhan S, Varghese M, Benegal V, et al. Prevalence and socioeconomic impact of depressive disorders in India: multisite population-based cross-sectional study. BMJ Open. 2019;9(6):e027250. doi: 10.1136/bmjopen-2018-027250 31253618 PMC6609075

[9] Gururaj G, Varghese M, Benegal V. National mental health survey of India, 2015-16: prevalence, pattern and outcomes. 2016.

[10] Girase B, Parikh R, Vashisht S, Mullick A, Ambhore V, Maknikar S. India’s policy and programmatic response to mental health of young people: a narrative review. SSM - Mental Health. 2022;2. doi: 10.1016/j.ssmmh.2022.100145

[11] Meghrajani VR, Marathe M, Sharma R, Potdukhe A, Wanjari MB, Taksande AB. A comprehensive analysis of mental health problems in India and the role of mental asylums. Cureus. 2023;15(7):e42559. doi: 10.7759/cureus.42559 37637646 PMC10460242

[12] Paul R, Muhammad T, Rashmi R, Sharma P, Srivastava S, Zanwar PP. Depression by gender and associated factors among older adults in India: implications for age-friendly policies. Sci Rep. 2023;13(1):17651. doi: 10.1038/s41598-023-44762-8 37848598 PMC10582097

[13] Gaiha SM, Taylor Salisbury T, Koschorke M, Raman U, Petticrew M. Stigma associated with mental health problems among young people in India: a systematic review of magnitude, manifestations and recommendations. BMC Psychiatry. 2020;20(1):538. doi: 10.1186/s12888-020-02937-x 33198678 PMC7667785

[14] Suresh K, Dar AA. Mental health of young adults pursuing higher education in Tier-1 cities of India: a cross-sectional study. Asian J Psychiatr. 2025;106:104447. doi: 10.1016/j.ajp.2025.104447 40088751

[15] Silva GFS, Fagundes TP, Teixeira BC, Chiavegatto Filho ADP. Machine learning for hypertension prediction: a systematic review. Curr Hypertens Rep. 2022;24(11):523–33. doi: 10.1007/s11906-022-01212-6 35731335

[16] Kourou K, Exarchos TP, Exarchos KP, Karamouzis MV, Fotiadis DI. Machine learning applications in cancer prognosis and prediction. Comput Struct Biotechnol J. 2014;13:8–17. doi: 10.1016/j.csbj.2014.11.005 25750696 PMC4348437

[17] Sattari M, Mohammadi M. Using data mining techniques to predict chronic kidney disease: a review study. Int J Prev Med. 2023;14:110. doi: 10.4103/ijpvm.ijpvm_482_21 37855011 PMC10580203

[18] D’Arrigo G, Gori M, Pitino A, Torino C, Roumeliotis S, Tripepi G. Statistical methods to assess the prognostic value of risk prediction rules in clinical research. Aging Clin Exp Res. 2021;33(2):279–83. doi: 10.1007/s40520-020-01542-y 32240502

[19] Zhang L, Wei R, Zhou J, Tan L, Che X, Zhang M, et al. Predicting depression and unravelling its heterogeneous influences in middle-aged and older people populations: a machine learning approach. BMC Psychol. 2025;13(1):395. doi: 10.1186/s40359-025-02691-3 40247342 PMC12004675

[20] Lee C, Kim H. Machine learning-based predictive modeling of depression in hypertensive populations. PLoS One. 2022;17(7):e0272330. doi: 10.1371/journal.pone.0272330 35905087 PMC9337649

[21] Park J, Baik J-H, Adjei-Nimoh S, Lee WH. Advancements in artificial intelligence-based technologies for PFAS detection, monitoring, and management. Sci Total Environ. 2025;980:179536. doi: 10.1016/j.scitotenv.2025.179536 40311342

[22] Lee C, Gates KM. Automated machine learning for classification and regression: a tutorial for psychologists. Behav Res Methods. 2025;57(9):262. doi: 10.3758/s13428-025-02684-5 40826202

[23] Mahamadou AJD, Rodrigues EA, Vakorin V, Antoine V, Moreno S. Interpretable machine learning for precision cognitive aging. Front Comput Neurosci. 2025;19:1560064. doi: 10.3389/fncom.2025.1560064 40452951 PMC12122752

[24] Kruschel S, Hambauer N, Weinzierl S, Zilker S, Kraus M, Zschech P. Challenging the performance-interpretability trade-off: an evaluation of interpretable machine learning models. Business Inform Systems Eng. 2025. doi: 10.1007/s12599-024-00922-2

[25] Arokiasamy P, Sekher TV, Lhungdim H, Dhar M, Roy AK. Study on Global AGEing and Adult Health (SAGE) Wave 2, India National Report. 2020.

[26] Ferrouhi EM, Bouabdallaoui I. A comparative study of ensemble learning algorithms for high-frequency trading. Sci Afr. 2024;24. doi: 10.1016/j.sciaf.2024.e02161

[27] Imani M, Beikmohammadi A, Arabnia HR. Comprehensive analysis of random forest and XGBoost performance with SMOTE, ADASYN, and GNUS under varying imbalance levels. Technologies. 2025;13(3):88. doi: 10.3390/technologies13030088

[28] Haruna U, Mohammed A-R, Braimah M. Understanding the burden of depression, anxiety and stress among first-year undergraduate students. BMC Psychiatry. 2025;25(1):632. doi: 10.1186/s12888-025-07069-8 40598169 PMC12220226

[29] Kundu S, Bakchi J, Al Banna MH, Sayeed A, Hasan MT, Abid MT, et al. Depressive symptoms associated with loneliness and physical activities among graduate university students in Bangladesh: findings from a cross-sectional pilot study. Heliyon. 2021;7(3):e06401. doi: 10.1016/j.heliyon.2021.e06401 33748473 PMC7969900

[30] Shatte ABR, Hutchinson DM, Teague SJ. Machine learning in mental health: a scoping review of methods and applications. Psychol Med. 2019;49(9):1426–48. doi: 10.1017/S0033291719000151 30744717

[31] Kessler RC, Berglund P, Demler O, Jin R, Koretz D, Merikangas KR, et al. The epidemiology of major depressive disorder: results from the National Comorbidity Survey Replication (NCS-R). JAMA. 2003;289(23):3095–105. doi: 10.1001/jama.289.23.3095 12813115

[32] Poongothai S, Pradeepa R, Ganesan A, Mohan V. Prevalence of depression in a large urban South Indian population--the Chennai Urban Rural Epidemiology Study (CURES-70). PLoS One. 2009;4(9):e7185. doi: 10.1371/journal.pone.0007185 19784380 PMC2748692

[33] Reddy VM, Chandrashekar CR. Prevalence of mental and behavioural disorders in India: a meta-analysis. Indian J Psychiatry. 1998;40(2):149–57. 21494462 PMC2965838

[34] Ma H, Cai M, Wang H. Emotional blunting in patients with major depressive disorder: a brief non-systematic review of current research. Front Psychiatry. 2021;12:792960. doi: 10.3389/fpsyt.2021.792960 34970173 PMC8712545

[35] Chekroud AM, Zotti RJ, Shehzad Z, Gueorguieva R, Johnson MK, Trivedi MH, et al. Cross-trial prediction of treatment outcome in depression: a machine learning approach. Lancet Psychiatry. 2016;3(3):243–50. doi: 10.1016/S2215-0366(15)00471-X 26803397

[36] Dwyer DB, Falkai P, Koutsouleris N. Machine learning approaches for clinical psychology and psychiatry. 2025. doi: 10.1146/annurev-clinpsy-032816 29401044

[37] Kirkbride JB, Anglin DM, Colman I, Dykxhoorn J, Jones PB, Patalay P, et al. The social determinants of mental health and disorder: evidence, prevention and recommendations. World Psychiatry. 2024;23(1):58–90. doi: 10.1002/wps.21160 38214615 PMC10786006

[38] Marufi N, Malekzadeh R, Naderi F, Garmabi M, Sharifnezhad A, Darrudi F, et al. Association of depression and anxiety with health-related quality of life in beginning Medical Sciences Student. Sci Rep. 2024;14(1):24515. doi: 10.1038/s41598-024-76503-w 39424991 PMC11489748

[39] Akhtar SN, Saikia N, Muhammad T. Self-rated health among older adults in India: Gender specific findings from National Sample Survey. PLoS One. 2023;18(4):e0284321. doi: 10.1371/journal.pone.0284321 37068072 PMC10109469

