# Supplementary material for: Depression prediction and key factors: A comparative analysis of logistic regression and machine learning models
Source: PLoS One. 2026 Aug 27;21(8):e0354668. doi: 10.1371/journal.pone.0354668 (PMC13521342; doi:10.1371/journal.pone.0354668)
Supplement: Supplementary file 1 [file pone.0354668.s001.pdf]

S1 Table: Hyperparameter Tuning for the ML models

**Model**	**Hyperparameters tuned**
Random Forest	mtry
SVM (RBF)	Cost (C), Sigma
KNN	k
Decision Tree	cp
Neural Network	size, decay
Ridge Logistic	lambda
XGBoost	nrounds, max_depth, eta, subsample, colsample_bytree
