# Supplementary material for: Depression prediction and key factors: A comparative analysis of logistic regression and machine learning models
Source: PLoS One. 2026 Aug 27;21(8):e0354668. doi: 10.1371/journal.pone.0354668 (PMC13521342; doi:10.1371/journal.pone.0354668)
Supplement: Supplementary file 2 [file pone.0354668.s002.pdf]

S2 Table: Supplementary performance metrics (precision, Hamming loss, and Jaccard score) for machine learning models used to predict depression in the study population.

Model	Precision	Hamming Loss	Jaccard Score
Random Forest	0.930	0.072	0.860
XGBoost	0.890	0.100	0.810
SVM	0.920	0.072	0.870
Logistic Regression	0.910	0.084	0.840
Bagging	0.910	0.079	0.850
Decision Tree	0.920	0.075	0.860
Naive Bayes	0.910	0.077	0.850
Ridge Logistic	0.920	0.072	0.860
Neural Net	0.880	0.105	0.780
KNN	0.910	0.072	0.860
